# “What I Wish I Would’ve Known before My HIV Diagnosis”: Qualitative Insights from Women Living with HIV to Inform HIV Prevention Strategies

**DOI:** 10.1089/apc.2025.0006

**Published:** 2025-05-08

**Authors:** Jessica L. Corcoran, Victoria McDonald, Alexandria L. Hahn, Randi Singer, Mirjam-Colette Kempf, Rebecca Schnall, Amy K. Johnson

**Affiliations:** ^1^ School of Nursing, The University of Alabama at Birmingham, Birmingham, AL, USA.; ^2^ Columbia University School of Nursing, New York, NY, USA.; ^3^ College of Nursing, University of Illinois Chicago, Chicago, IL, USA.; ^4^ School of Public Health, University of Alabama at Birmingham, Birmingham, AL, USA.; ^5^ Heersink School of Medicine, University of Alabama at Birmingham, Birmingham, AL, USA.; ^6^ Mailman School of Public Health, Columbia University, New York, NY, USA.; ^7^ Ann & Robert H. Lurie Children’s Hospital of Chicago, Chicago, IL, USA.; ^8^ Feinberg School of Medicine, Northwestern University, Chicago, IL, USA.

**Keywords:** HIV prevention, women living with HIV, qualitative analysis, risk reduction strategies

## Abstract

In 2022, cisgender women accounted for one in five HIV diagnoses in the United States. Existing structural, social, and behavioral factors impede women’s engagement with HIV prevention strategies including Pre-Exposure Prophylaxis (PrEP) access, condom usage, and uptake of HIV testing. This study explores the perspectives of women living with HIV (WLWH) about factors that may contribute to HIV acquisition and their advice for women who may benefit from HIV prevention strategies. We conducted semistructured interviews with 40 WLWH who were diagnosed on or after January 1, 2000. Interviews were conducted via Zoom and lasted 45–60 min. Interviews were professionally transcribed, coded, and analyzed to identify themes. Guided by the AIDS Risk Reduction Model, this study uses qualitative findings to describe the steps for recognizing and reducing HIV vulnerabilities. The analysis revealed three themes: (1) recognizing HIV risk for oneself and partner, (2) commiting to decreasing HIV risk, and (3) enacting HIV risk reduction strategies. After recognizing the personal and partner characteristics associated with increased HIV risk and committing to reducing that risk through self-love, relationship assertiveness, and boundary setting, women will be better prepared to enact risk reduction strategies. The four most commonly discussed strategies by WLWH included HIV testing, condom usage, PrEP, and avoiding drug use. This study highlights the importance of understanding the experiences of WLWH to inform effective HIV prevention strategies. Insights from these women emphasized the need for increased awareness, empowerment, and accessible resources to support HIV risk recognition and reduction among women.

## Introduction

In 2022, cisgender women, hereafter referred to as women, accounted for 1 in 5 new HIV diagnoses (19%) in the United States, with heterosexual contact being the primary mode of HIV transmission among women (84%).^1,2^ Despite overall declines in HIV incidence, racial disparities remain, with Black and Latina women together representing 73% of new HIV diagnoses compared with White women who represent 22% of new HIV diagnoses.^2^ Additionally, there is regional variation in HIV incidence rates, with the US South being recognized as the epicenter of the HIV epidemic within the United States.^1^ In 2022, 53% of HIV incident cases occurred in the South, 21% in the West, 13% in the Northeast, and 13% in the Midwest.^2,3^ To further achieve declines in HIV incidence rates, reaching women in communities with high HIV incidence rates and increasing awareness among women about HIV prevention strategies is necessary.^4^ We interviewed women living with HIV (WLWH) to understand the factors they perceived as contributing to their HIV diagnosis and to gather their advice to women who could benefit from HIV prevention strategies.

Existing structural, social, and behavioral factors impede women’s engagement with HIV prevention strategies.^4,5^ Structural barriers such as poverty and lack of access to health care often impede women’s ability to access HIV prevention services, including HIV testing, Pre-Exposure Prophylaxis (PrEP), and education.^4,6^ These challenges are further exacerbated by gender dynamics, with many women reporting difficulties negotiating HIV prevention measures—like condom use or partner HIV testing—due to concerns about partner trust or fear of intimate partner violence.^7,8^ Moreover, systemic inequalities, including racism, income disparities, interpersonal violence, and sexism, create compounding vulnerabilities for Black and Latina women, who are significantly overrepresented among WLWH.^5,7^ These women often live in communities with a higher prevalence of HIV, increasing the likelihood of exposure.^5,7^

Behavioral factors combined with insufficient HIV vulnerability awareness further complicate prevention efforts for women.^9,10^ Studies indicate that women often perceive themselves as not at risk for HIV, leading to lower uptake of prevention strategies such as condoms and PrEP.^10–15^ Further, societal stigma around HIV and sexual health impedes access to comprehensive sexual health education and discourages women from seeking testing and discussing prevention strategies with health care providers.^7,11,13,16^ Additionally, to enhance PrEP uptake among women, provider training is an important component.^17^

A meta-analysis of HIV risk reduction interventions for Black women supports the inclusion of intervention components such as increasing women’s STI/HIV knowledge, condom-use self-efficacy, and facilitating partner communication about sex and condom use.^9^ Additionally, the most effective interventions addressed personal empowerment, provided skills training in condom use and negotiation of safer sex, and used role-playing to teach negotiation skills.^9^ Together, these intervention components have shown to influence a woman’s perception of risk and ability to commit to decreasing risk behaviors, which are critical to enacting HIV risk reduction strategies.

This article seeks to contribute to the literature by qualitatively exploring and documenting the perspectives of WLWH on factors they perceived as contributing to their HIV diagnosis including limited awareness of HIV susceptibility and the influence of individual and partner characteristics that may have contributed to HIV acquisition. Additionally, this article highlights recommendations by WLWH for current and future HIV prevention, focusing on enhancing interventions aimed at women in the United States.

## Methods

### Overall study design

This study is a qualitative analysis of semistructured interviews conducted as part of the AWARE study^18^ (R01AI172469; MPIs: Schnall/Johnson/Kempf). In Aim 1 of the AWARE study, we explored recommendations for recruitment and retention strategies for developing a national cohort of women who could benefit from HIV prevention.^18^ While the primary focus of the interviews was on recruitment and retention for the longitudinal cohort study, additional topics such as experiences and challenges living with HIV and behaviors that may increase HIV exposure were discussed. Additionally, participants shared advice for women who could benefit from HIV prevention and offered their thoughts on what they wished they would have known prior to acquiring HIV. This study adheres to the Consolidated Criteria for Reporting Qualitative Research guidelines.^19^

### Recruitment and inclusion criteria

For the AWARE study, participants were recruited via convenience sampling through online advertisements on social media and direct outreach in clinical settings. In-depth interviews and focus groups were conducted with three groups: (1) women who could benefit from HIV prevention, (2) women diagnosed with HIV on or after January 1, 2000, and (3) experts in HIV prevention and treatment, including advocates and members of community advisory boards. Initial eligibility screening was conducted via REDCap, followed by confirmatory screening over the phone prior to scheduling interviews. All participants provided informed consent before their interviews, and their identity was confirmed to prevent fraud.

We analyzed semistructured interview data from 40 WLWH (Group 2). Participants in Group 2 were aged 14–54 years; assigned female sex at birth; identified as female; understood and could read English or Spanish; living within the United States and its territories; and diagnosed with HIV on or after January 1, 2000.

### Data collection and preparation

Preceding the interviews, participants completed a demographic survey administered via Qualtrics. The survey collected data on age, race, ethnicity, sexual orientation, marital status, living arrangements, education level, income, and health insurance status. The survey also included questions about the participant’s age at the first sexual encounter, their HIV and STI testing history, and their likely route of HIV acquisition.

After the survey, semistructured interviews were conducted using an interview guide developed and piloted with representatives from a nonprofit organization focused on women and HIV. Interviews were conducted via Zoom and lasted 45–60 min. Interview facilitators had a range of qualitative experience and included both master’s and PhD prepared staff. All interview facilitators identified as female. Participants received a $50 Amazon e-gift card after completing the interview. All interviews were audio recorded and professionally transcribed. Data were managed using Dedoose, a cloud-based flexible and adaptive qualitative analysis program.

### Conceptual framework

**FIG. 1.**
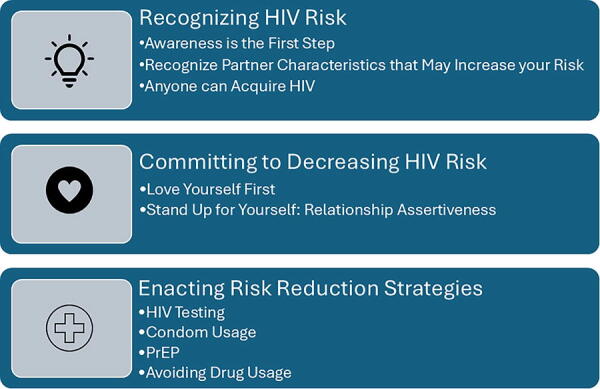
Modification of the the AIDS Risk Reduction Model.

An adaptation of the AIDS risk reduction model (ARRM)^20,21^ guided the data analysis (Fig. 1). Originally, this model was developed by Catania^21^ to understand an individual’s behavior in reducing their risk for HIV infection. The ARRM is composed of three sequential stages: (1) recognizing and labeling one’s behaviors as risky, (2) making a commitment to reducing high-risk behaviors, and (3) taking specific actions to reduce risks, such as consistent condom use and HIV testing. The model highlights the importance of cognitive, emotional, and social influences on behavior change while also accounting for barriers such as stigma, denial, and misconceptions about HIV transmission.^21^ This model has been widely employed in interventions promoting HIV prevention, particularly among priority populations, offering a structured approach to address both individual and environmental factors that influence safer sex practices.^20,22^ This model is useful because it can illuminate the additional factors necessary when developing HIV risk reduction interventions from the perspective of WLWH. This model continues to inform public health initiatives by providing a framework for designing and implementing effective HIV prevention strategies.

### Data analysis

This analysis builds on the coding framework established in the AWARE study interviews which employed a rapid content analysis approach.^23^ A codebook was developed, including a code tree that all coders reviewed and refined. Coders were divided into three pods and assigned transcripts within participant groups 1, 2, or 3. A PhD-level coding lead was identified for each pod; the lead coder reviewed the primary code applications, ensured code application agreement, and resolved any coding disagreements within the pod. Most divergences within pods occurred due to omission and were quickly rectified to 100% agreement. Throughout the coding process, the lead coders (J.C., A.J., and R.S.) met weekly to discuss code application and ensure consistency of application across participant groups.

For this project, we analyzed interview data from WLWH (Group 2) using Braun and Clarkes’ six steps of thematic analysis.^24^ The six steps of thematic analysis, as described by Braun, Clarke, Hayfield, and Terry, are: (1) familiarize yourself with the data, (2) generate initial codes, (3) search for themes, (4) review themes, (5) define themes, and (6) write-up analysis.^24^ Two researchers (J.C. and V.M.) with backgrounds in research and qualitative methodology conducted the coding and analysis. An updated coding frame was developed with new codes to explore advice for women who could benefit from HIV prevention and reflections from WLWH. These codes and the subsequent themes were developed based on the ARRM. One researcher (J.C.) updated the coding frame and applied new codes to relevant excerpts from the interview data. Then, the primary coder discussed with the secondary coder (V.M.) until intercoder agreement was reached.^25^ This process also involved regular discussions to refine the coding framework and integrate the new codes with those previously established. The researchers reviewed and compared the coded data to identify patterns and relationships––grouping related codes into broader categories and refining them to identify overarching themes.^24,25^ Regular discussions ensured that the themes accurately represented the data. To ensure the credibility and dependability of our interpretations, we conducted peer debriefings with a broader group of researchers to validate our findings. We maintained an audit trail to document the coding decisions throughout the analysis.^25,26^

### Ethical considerations

The AWARE study was approved by the Institutional Review Board at Columbia University (IRBAAU2650). Lurie Children’s Hospital of Chicago (IRB23-5921) and the University of Alabama at Birmingham (IRB-300010515) were approved via an IRB reliance agreement with Columbia University.

## Results

Table 1 illustrates the demographics of the 40 WLWH in this study. Guided by the ARRM,^16^ the analysis revealed three themes: (1) recognizing HIV risk, (2) committing to decreasing HIV risk, and (3) enacting HIV risk reduction strategies. The risk reduction strategies from WLWH included the recommendations to get tested for HIV, use condoms, use PrEP, and avoid drug usage.

**Table 1.** Characteristics of Study Sample

WLWH (*n* = 40)	
Age at enrollment	
Average years (SD)	46.5 (12.8)
Age at First Sexual Encounter	
Average years (SD)	15.6 (3)
Missing	1
Race	
Black/African American	82.5 (33)
White	17.5 (7)
Ethnicity	
Hispanic	8.1 (3)
Non-Hispanic	91.9 (34)
Missing	3
Sexual Orientation	
Heterosexual	82.5 (33)
Asexual	2.5 (1)
Bisexual	10 (4)
Not reported	5 (2)
Marital Status	
Divorced with no current partner	15 (6)
Have a partner not living with you	12.5 (5)
Living with partner	10 (4)
Married	15 (6)
Separated with no current partner	5 (2)
Single (Never been married)	32.5 (13)
Widowed with no current partner	10 (4)
Living Status	
Living alone	42.5 (17)
Living alone with children	20 (8)
Living with friends/family	12.5 (5)
Living with spouse/ partner and children	7.5 (3)
Living with spouse/partner	15 (6)
Other	2.5 (1)
Education	
Completed 8th grade	10 (4)
Completed GED	5 (2)
Graduated High School	35 (14)
Some College	32.5 (13)
College graduate	10 (4)
Attended graduate school	7.5 (3)
Annual Household Income	
$0–$4999	25.6 (10)
$5000–$19,999	28.2 (11)
$20,000–$49,999	30.7 (12)
$50,000+	15.4 (6)
Missing	2.5 (1)
Health Insurance	
No	2.5 (1)
Yes	97.5 (39)
Health Insurance Type^*^	
Private Insurance	57.5 (23)
Public insurance (Medicare, Medicaid)	57.5 (23)
No insurance/ Uninsured	2.5 (1)
Had STI Past Year	
No	67.5 (27)
Yes	32.5 (13)
Perceived Mode of HIV Infection^*^	
Needle sharing with HIV positive person	7.5 (3)
Sex with an HIV positive person	80 (32)
Rape	5 (2)
Victim of violent crime	2.5 (1)
Don’t know	7.5 (3)

*Notes.* Data are % (*n*).

^*^ Not mutually exclusive.

SD, Standard deviation; WLWH, women living with HIV.

### Recognizing HIV risk

The first theme describes how WLWH emphasized the importance of women recognizing the potential for HIV exposure and encouraging greater awareness of personal and partner behaviors and characteristics that may contribute to HIV acquisition. The three subthemes include *Awareness is the First Step*, *Recognize Partner Characteristics that May Increase your Risk,* and *Anyone Can Acquire HIV*.

#### Awareness is the first step

Many WLWH discussed the importance of increasing knowledge and awareness of HIV risk factors. Several women discussed how the information about HIV risk factors exists, yet awareness is lacking. One woman said, “There’s a whole bunch of like websites and videos about such things, right? I think just the awareness is lacking Int 42.” Another woman, when she was asked for her advice for women potentially exposed to HIV, emphasized, “Education, education, education. There’s too much information that’s out there that’s on Google, that I know everybody gets on, for you not to know. Point blank Int 30.” Another participant highlighted the need for education, particularly from reliable sources. “Try to seek support from organizations or resources that actually focus on women’s sexual health and HIV prevention. And educate yourself to learn more and not just listen to what people tell you. Like learn from reliable sources Int 13”. Another participant recognized the lack of awareness, especially for young people and her advice was to talk to people when they are younger to make them more aware.

“Talk to them [women who could benefit from HIV prevention] and tell them it’s a lot of it [HIV] out here. And like, it’s a lot of the younger generation that’s falling into that… Just talking about you have to protect yourself, no matter how old you are or how young you are, because there’s a lot of young people now getting it Int 15.”

Three participants discussed the importance of providing information about preventive measures and HIV education in school settings. All participants discussed dissatisfaction with the education they received in schools. When asked what might have prevented her HIV diagnosis, one participant highlighted how comprehensive sex education in schools must begin at a younger age.

More information and awareness, just more talked about, like it is now….But definitely, more preventative measures in the schools. Yes, give it [sex ed] often in the schools at a much younger age than when we started receiving that knowledge Int 35.

Another participant echoed the need for expanded sex education in schools, noting that regional variations can create barriers in accessing school-based sex education.

I mean, there needs to be education about it. I think that the gay community is very educated about it, but we [women] kind of, especially in the south, we can’t talk about sex in schools; we can’t talk about it, you know, anywhere. So, it’s getting that information out Int 17.

Another participant echoed the need for more discussions in schools, but added that alongside school-based education, she also wished her parents had talked with her about sex and its associated risks.

“I wish they would have talked about it [HIV] more in school, when I was going to school. I wish my parents would have talked about it more, too. I was kind of clueless on the activities that were going on out there; what people were doing and what they were getting in. Stuff like that Int 27.”

In addition to the need for increased education about HIV risk factors and preventive measures, participants also emphasized the importance of education to remove stigma and shame surrounding HIV and sexual behavior. One participant noted how education is the best defense against HIV, as it can facilitate both prevention and destigmatization.

“Get educated. That’s the best defense. I really believe that that’s the best defense. Because once you get education, then education leads you to things like PrEP, condoms, safe sex, things like that nature. It’ll rid yourself of stigma, all of that stuff Int 44.”

Another participant echoed the need for awareness without shame, highlighting that recognizing the risk and increasing awareness are essential first steps in protecting oneself.

“I think…either foundations or community, they should create awareness about HIV. And like try creating ways to make it not look shameful thinking about it, discussing about it. So, awareness is the first step. Like reaching out to people who don’t know the risk they are into and making this discussion not shameful to any woman Int 10.”

Recognizing the impact of shame and stigma surrounding sexual health, one participant emphasized that discussions about sexual health should not feel immoral. She said, “So, talking about HIV and STI or sexual health, it shouldn’t like feel like you’re immoral Int 10.” Another woman echoed the thought around stigma and HIV.

“It’s the stigma of it. I don’t like the stigma of it…when I was in high school after I learned about HIV, I was afraid to be around anybody who had HIV…So, I just wish the stigma around it [HIV], there was less Int 46.”

Overall, WLWH stressed the importance of raising awareness about risk factors associated with HIV. They highlighted that schools are underutilized resources that should be capable of offering destigmatized sex education to improve knowledge and awareness.

#### Recognize partner characteristics that may increase your risk

In addition to discussing awareness of HIV within their communities and personal risk factors, WLWH also reflected on partner characteristics that they attributed to their HIV diagnosis. The most commonly discussed partner characteristics included recent incarceration, identifying as bisexual or gay, and engaging in promiscuous behavior. When asked what she wished she had known prior to acquiring HIV, one participant shared that her partner had been in prison. She believed that his recent incarceration contributed to her diagnosis.

“Yeah, I knew it [HIV] was out there, but I didn’t think I would ever come across it and never thought that I would be the one. Mine was a guy who was in prison. And so, just the fact that a lot of people come from prison, men do things in there that they’re not going to tell you about. And just to be careful of anybody, anybody. Like I said, it could be your…boyfriend, just being more aware of it, being talked about more Int 40.”

Another participant mentioned similar hindsight, noting that the partner she believes she acquired HIV from had recently been to prison. She stated, “I wished I would have known more about it [HIV]. When I found out what I had [HIV], I found out he had been in prison, and that was never told to me until after I got it Int 19”. In addition to recent incarceration, WLWH also discussed their partners’ sexual orientation as a possible risk factor.

“I would say know the person that you’re with. Because when I think back on it, there were signs. The man that infected me, he was bisexual. I never knew that. It never dawned on me, until it was told to me Int 26.”

Another participant echoed this thought about knowing more about her partner’s sexual behavior. When she was asked what could have prevented her HIV diagnosis, “Probably prevention. Had I known…so, back then, that there were a lot of bisexual men. And yeah, that would’ve helped me. Int 39.” She also emphasized the importance of educating women about the risks associated with having multiple and/or bisexual partners. Her advice for women was, “Well, just talking to them [women] about having multiple partners, unprotected sex, bisexual partners Int 39.” Another woman discussed the importance of knowing if a male partner is sleeping with multiple women. Reflecting on her own experiences, she said

“I think because of my lifestyle [injection drug use], I was always aware, you know. But I don’t think that I got it while I was using [drugs], you know. Women need to know their partners. And act accordingly, which I knew he was a ho Int 32.”

The importance of recognizing partner-related HIV risk factors was a recurring theme in the discussions with WLWH.

#### Anyone can acquire HIV

When one WLWH was asked what advice, she would offer women who could benefit from HIV prevention, she stressed that anyone can contract HIV, suggesting all women who are HIV negative would benefit from HIV prevention.

“No matter how they [person living with HIV] look, you never know who has it. It don’t have no face. That’s the thing. Because the person I never thought they would have nothing like that [HIV]. From the way they was and the way they dressed, and in the way they kept their self Int 40.”

Another WLWH discussed similar thoughts and reflected on partner trust and naivety. When she was asked what could have prevented HIV, she said, “Not being as trusting of people. And always we should have went and get tested together before I even laid down with him. But I was young, naïve. He was a little bit older. I was trusting Int 31.” Another participant discussed that anyone is susceptible to HIV. Her advice for women who could benefit from HIV prevention was, “My last thought would be do not think you are unique. Everybody is subject to this disease…My thing is stop thinking you’re so unique that you can’t contract this virus Int 38.”

Several WLWH expressed that many women believe they are not at risk for HIV. When one woman was asked about the benefits of a study aimed at exploring HIV risk factors in women, she highlighted that raising awareness of HIV risk itself would be a significant benefit.

“Well, people will be more self-aware, and more self-aware of other people around them, and just their situations. I think it will help people not be so, like, oh, it could never happen to me, kind of attitude, because it can happen [HIV diagnosis]. It can happen to the most richest, nicest person in the world, it can happen. It’s scary. And it’s sad that it’s like that, but I mean that’s just the reality of it Int 18.”

Another participant reiterated the sentiment that women do not feel susceptible to HIV. She explained, “You see it [HIV] on TV… But you’re still thinking it’s not going to happen. You know? I ain’t got to worry about that. That’s over there somewhere Int 32.” According to the AARM, recognizing the risk for HIV is the first step. WLWH highlighted the importance of being aware of the personal and partner characteristics that may increase risk, as well as acknowledging one’s own susceptibility to HIV.

### Committing to decreasing HIV risk

The second step, after recognizing the risk of HIV, is committing to reducing that risk.^20,21^ For WLWH, this commitment to decreasing HIV risk presented through loving yourself, standing up for themselves, and practicing relationship assertiveness. The WLWH felt that despite the cultural shift enabling women to be more assertive, more progress is needed. Equipping women with the cognitive and behavioral tools to enact and enforce boundaries is essential to ultimately reducing HIV risk. This theme has two subthemes *Love Yourself First and Stand Up for Yourself: Relationship Assertiveness.*

#### Love yourself first

WLWH discussed the importance of self-love as a critical step in mitigating risk of HIV. One participant explained that loving oneself must come first; stating, “But you know… I called myself in love. Yeah. I said we need to love ourselves. Love ourselves first, you know Int 32.” Another woman echoed this sentiment, emphasizing that even if you think you are in love with someone, you must prioritize self-love.

“So, definitely put yourself first. In every situation, protect yourself at all costs no matter what. And no matter how mad somebody gets, don’t worry about it, because they’re just not it. So, protect yourself at all costs, no matter what it is, whether it’s love and all those feelings, it covers up the realness. And sometimes you have to take a step back and just deal with what is real and what is the love part. But, you know, I had to go through that Int 35.”

Another woman expressed that, in her opinion, if she had loved herself more, she might not have acquired HIV. She stated, “I think that…me loving myself more inside and saying, ok, this is enough. No matter what, don’t go back Int 39.” She further expounds on this, saying, “Well, I just wish I had loved myself more and realized that I should’ve left sooner. That’s what my issue is. And maybe…I wouldn’t even be in this situation [acquired HIV] had I left sooner Int 39.” The concept of loving oneself enough to recognize risk factors or red flags in a relationship and take action, such as leaving, was an important theme discussed by WLWH.

#### Stand up for yourself: Relationship assertiveness

Building on the theme of self-love, another participant discussed the importance of self-esteem and standing up for oneself as essential advice for women as a facilitator for HIV prevention.

“You have to stand up for yourself. You can’t rely with nobody else to give you self-esteem. I can’t give you self-esteem. That has to come from within you. Or, you can mimic what you want from me into yourself, until you’re strong enough to stand up on your own. But you have to mold it to fit you, not the way I do it. Because everything is different, your approach Int 21.”

Another participant discussed the importance of assertiveness in relationships. When asked what advice she would give to women, she referenced the education she received about being more assertive and initiating conversations around setting boundaries.

“I did a fellowship with Planned Parenthood, and they have a whole section on like consent, and conversations about condoms, and how women need to like be taught to be assertive in that area, and how we go on birth control just so we don’t have to have these conversations. And so, to teach women how to take responsibility and accountability and be able to be assertive, because it is your body, and it will affect you forever. And that guy is probably not going to be there forever. But you’re stuck with you. So, whatever him or the boy after him, or whoever, gives you—I mean, you’re stuck with HIV. (laughter) And I might never talk to him again, but I will forever have HIV because of him. And just the knowledge that like women are not taught to be vocal about sex and about protecting themselves, and about our bodies, right? We’re to smile and be nice to people, and hug whoever; and we’re not taught to like speak up and set boundaries. So, to be able to teach women how to actually have those conversations where they’re like no, like you need a condom… I guess to teach them how to do that Int 42”.

“So, I feel like that’s some of the ways that women that are not positive should go about preventing. You know, being more—a little more assertive. Like put your foot down, you know. Don’t be a little wimpy wimp; and then you’re sorry when it’s too late. You know what I mean? Int 45.”

In addition to relationship assertiveness, several women emphasized the importance of open conversations with partners about boundaries. One woman said, “You know just having conversations, real serious conversations with your partner. Like, hey, you know listen, I know we’ve been together. But I think we need to talk about using protection… We need to talk about some barriers Int 7.” Another participant noted a societal expectation, highlighting that both women and men are now more empowered to speak openly about boundaries and protecting themselves.

Now, a lot of women are being vocal about, ‘I can’t lay down with you unless I know that you’ve been tested.’ Back then, that was unheard of. That was unheard of altogether. So, women being able to take charge. Men being able to take charges nowadays, too. “Hey. I’m not going to lay with you unless I know that you’ve been tested. I’m free and negative of STIs. I want to make sure that you are Int 30.”

Another WLWH, stated the importance of boundaries, expressing that she might not have acquired HIV if she had established boundaries earlier in life. She said, “Like if I had boundaries in my twenties, my life would be way different Int 42.” Overall, WLWH stressed the significance of self-love and assertiveness in relationships as critical strategies for reducing the likelihood of acquiring HIV.

### Enacting HIV risk reduction strategies: “if you care about life and you wanta live, you have to do these things”

After recognizing the personal and partner characteristics associated with increased HIV risk and committing to reducing that risk through self-love, relationship assertiveness, and boundary setting, women will be better prepared to enact risk reduction strategies. The four most commonly discussed strategies by WLWH included *HIV testing, condom usage, PrEP, and avoiding drug use.*

#### HIV testing

The most commonly recommended risk reduction strategy mentioned by WLWH was getting tested for HIV. One participant described testing as the initial step in taking care of sexual health, stating, “The first step, I believe, would be getting tested. That way you will know if you need to get in care or take preventative measures Int 32.” Another participant highlighted that testing should be the first step and a routine practice. Her advice for women was, “I would say get tested. Test yourself every three months. If you’re very sexual, every month. But stay on top of those two things; wrap it up and test Int 31.” A third participant also discussed the importance of testing but included drug use as a risk factor alongside sex; she said, “If you’re sexually active or any type of on drugs stuff, you need to go get tested, because if you care about your life; if you care about life and you wanta live, you have to do these things Int 46.”

In addition to testing themselves, WLWH emphasized the importance of ensuring their partners are also tested. One participant shared,

“And so, I would tell any woman, get the test. Be tested. Test your partner. If it’s somebody that you’re going to be laying down with and be intimate with, that means you’re leaving yourself and your body vulnerable. So, you know you need to know. If they care enough about you, they’re not going to have any objections to being tested for HIV. Because they need to know their health status as well Int 26”

Another participant also recommended knowing their partner’s status. When asked what advice she would give to women, she said, “Know more. Know your status, know your partner’s status. That’s itInt 27.”

A third participant stressed the importance of being aware of a partner’s status and attributed her unawareness as a factor that led to her acquiring HIV.

“I’ll say knowing fully well that HIV is a communicable disease and you just have to make sure you protect yourself just like, for example, I got infected by a partner. I was so careless and I never knew his status. I had unprotected sex and definitely I was infected. That was a very, very risky practice. I will say it’s very important to know the status of your partner Int 11.”

#### Condom usage

Condom usage was the second most commonly recommended strategy. Many women emphasized the importance of protected sex. When one participant, was asked what advice she would offer women, she said, “It would be to always wrap it up, because you never know. You can’t tell a book by its cover. Just because it looks good don’t mean it’s good. …so, my advice would always be to wrap it up Int 31.” Another participant echoed this sentiment, stating “My advice is just always making sure that you have protected sex, irrespective of the partner, irrespective of anything…just make sure you’re having protected sex Int 12.” Both participants emphasized the universality of the recommendation, reflecting the belief that protection should be used consistently and without exception. They highlighted that no situation or partner is without risk. Another participant added, “I am going to say this tongue in cheek, but just use the freaking condoms. I think of all the bullets I dodged Int 16.” Her reflection underscores the risks she took in the past by assuming she had a low likelihood of acquiring HIV and other sexually transmitted infections. This sentiment was echoed by several women, but the struggles women face in negotiating condom usage with their partners were also discussed. One participant shared her experience, saying,

“If you have sex, you know you need to have protected sex. And you need to be with a partner who understands and goes along with your wishes. Yeah. Men will tell you; I can’t wear them. Well, we can’t have sex. Period. That’s the bottom line. You know? They tell me, I’m not scared of you. That’s not why I want protection. I’m protecting me. It ain’t for you; this is for me. I don’t know what you got Int 32.”

This quote reflects the difficulties some women face when negotiating condom usage with partners who resist. The participant acknowledges that her motivation for protection is rooted in self-protection, not in catering to her partner’s preferences. This dissonance underscores a broader issue where women, in particular, must assert their health priorities in the face of resistance from male partners.

#### PrEP

Another common prevention strategy mentioned by WLWH was PrEP. One participant disclosed that her sister was taking PrEP at the time the participant was diagnosed with HIV. She expressed frustration at not knowing about PrEP, wishing her sister would have informed her about how to protect herself. When asked what could have prevented her HIV diagnosis, her response was, “PrEP! Why did she [the participant’s sister] not tell me she was on PrEP? I would’ve then got on PrEP! Like I said, you need to [get on PrEP]…especially like when you’re in college Int 20.”

Another participant similarly expressed regret over not knowing about PrEP before her diagnosis. She said, “I wouldn’t have been so careless. If I would have known about PrEP, I would have been taking it Int 36.” Two other women described PrEP as a recommendation for women with an increased likelihood of acquiring HIV; one said, “Basically, the PrEP drug…where you could safely, you know, just so to be on the safer side int 14’. The other said, “And women at higher risk should also consider PrEP Int 13.”

Overall, these quotes highlight the urgent need for increased education, communication, and involvement from health care providers to facilitate awareness and accessibility of PrEP. This is especially important for young adults and individuals in high-incidence settings such as college campuses. Without knowledge of and access to these treatments, vital prevention measures remain underutilized.

#### Drug usage

Another important but less commonly discussed theme was drug use. When one participant was asked what she would tell women, she said, “Just don’t use people’s needles Int 2.” Another participant disclosed how her drug use in conjunction with a sexual assault led to her acquiring HIV. She stated:

“One thing, not getting on drugs. That was the main thing. I’ve given up on who did it. When you go there…who did I get it from. That part will drive you crazy, so you have to get past that part. I do suspect a person because I was with somebody…as much as I could be committed because I was using drugs. I went out with this guy and the first time we used condoms. I was on crack and he was snorting cocaine. The first time we had sex, we used a condom, but then he insisted on sex again, and he got very violent with me and just forced himself on me because he felt he didn’t get his money’s worth the first time. A few years later when I found out I was positive and I [went to the HIV clinic]. Guess who was sitting up in the [HIV clinic]. He was. Int 38.”

WLWH discussed the importance of HIV testing, condom usage, PrEP, and avoiding drug use as risk reduction strategies. They emphasized the difficulty and the importance of including partners in testing and condom negotiations. According to the AARM, HIV risk reduction strategies are not commonly enacted until people are aware of their risk and susceptibility to HIV and are committed to reducing that risk.

## Discussion

This study explored insights of WLWH regarding advice for mitigating HIV risk and insights they wish they had known prior to their diagnosis. Understanding their perspectives of the factors that they perceived contributed to their HIV diagnosis provides valuable guidance for developing interventions aimed at preventing HIV among women.

This study highlights the importance of recognizing HIV risk factors and susceptibilities through increased education on personal and partner characteristics that may elevate women’s HIV vulnerabilities. Specifically, the findings about partner incarceration as a risk factor align with prior literature indicating that women whose partners are released from incarceration are more vulnerable to HIV acquisition. This risk is compounded, by low rates of condom use, lack of HIV testing, and limited awareness about prison-related risks for HIV transmission.^27^ Additionally, disparities in incarceration among Black and Latino men may exacerbate HIV incidence among Black and Latina women through increased risk behaviors of their formerly incarcerated partners.^27–29^ Recently, there has been an alarming trend, from 2010 to 2022, HIV rates declined by 25% among White individuals and 29% among Black individuals, while increasing by 12% among the Latino population.^1,30^ This highlights a significant disparity in HIV trends across racial and ethnic groups in the United States.^30^

After recognizing HIV risk factors, the next step according to the AARM is committing to reducing that risk. The WLWH in this study described this commitment through self-love, relationship assertiveness, and setting boundaries with partners. Despite existing barriers, these findings about the importance of assertiveness and effective communication are consistent with existing literature, which identifies these skills as essential to enhancing the effectiveness of HIV prevention interventions.^9^

Decreasing HIV risk requires social and emotional skills, including the ability to navigate difficult conversations with a sexual partner.^31^ Prioritizing oneself and practicing self-love are foundational skills that may not be easily taught but could be fostered through peer support groups that enhance self-esteem and personal empowerment.^32,33^ Additionally, role-playing partner conversations could increase the perceived ability to have future difficult partner conversations.^31,34^

Finally, risk reduction strategies that are proven to reduce HIV acquisition include regular HIV testing, consistent condom use, taking PrEP as prescribed, and avoiding drug usage.^9–12,35^ These strategies, supported by prior research, remain fundamental components of effective HIV prevention efforts and should be leveraged to reduce risk of HIV exposure. In addition to testing oneself, the WLWH discussed the importance of partner HIV testing. This is a very effective strategy; however, implementation and uptake of partner testing, especially in groups with higher HIV incidence (i.e., recent incarceration or history of drug usage) has been suboptimal. Partner testing as a risk reduction strategy requires further exploration with input from WLWH and partners on how best to engage women and partners in testing. Regular testing enables individuals to identify infections early, facilitating timely treatment and reducing the likelihood of transmission. The inclusion of partner testing in prevention interventions is especially significant, as it not only informs individuals about their partner’s serostatus but also fosters open communication about sexual health within relationships. This approach is particularly important in contexts where HIV risk is elevated due to partner behaviors, such as post-incarceration transitions or engagement in high-risk sexual or drug-related activities. Previous studies emphasize that increasing accessibility to partner testing and integrating it into routine health care visits can normalize the practice and reduce stigma. Moreover, partner testing is an integral component of a holistic risk reduction strategy, working synergistically with other preventive measures, such as consistent condom use and adherence to PrEP.

### Study limitations

This study has several limitations that should be considered when interpreting the findings. First, the use of convenience sampling may limit the generalizability of the results, as the sample may not fully represent the diverse experiences of all WLWH across the United States.^36^ Additionally, recruiting participants primarily through online platforms and clinic settings may have favored those who have greater access to technology and health care, potentially underrepresenting WLWH who face greater systemic or structural barriers. The self-reported nature of the data introduces the possibility of recall bias or social desirability bias,^37^ particularly given the sensitive topics of HIV transmission and sexual behavior. Finally, the thematic analysis was guided by the ARRM, which may have influenced the identification and interpretation of themes, potentially limiting the exploration of factors outside of the model’s framework.

### Recommendations for future research

Our findings suggest that HIV prevention efforts targeting women vulnerable to HIV acquisition should consider the content and delivery of educational messaging to increase awareness of HIV susceptibility. Specifically, future interventions should focus on helping women recognize their susceptibility to HIV. This may occur through leveraging community venues or social media platforms where WLWH can discuss their stories as trusted messengers. Learning from someone with lived experience of HIV may increase a woman’s perception of susceptibility, enabling her to move into the second phase of the ARRM, where she commits to reducing her risk. Additionally, incorporating sexual health and PrEP information into ob/gyn and primary health care clinic visits for women in areas with high incidence, could be an effective prevention strategy to increase women’s understanding of their vulnerability to HIV and the subsequent acceptability of PrEP.^38,39^

Many existing studies have primarily focused on decreasing HIV sexual risk behaviors (e.g., limiting sexual partners, initiating condom usage, and PrEP) without fully considering the upstream social, emotional, and educational components, like stigma reduction, that are critical for successful implementation of these HIV risk reduction strategies.^16,40,41^ These upstream factors play a key role in enabling women to act on HIV prevention tools effectively. Suggestions for future intervention development include emphasizing strategies to raise women’s awareness of HIV, enhance perceptions of susceptibility to HIV, and empower women to confidently advocate for and protect their sexual health.

We acknowledge that such interventions will be time, cost, and labor-intensive, and thus, a critical area for future research is to identify women, who would benefit most from these targeted HIV prevention interventions.^18^ While effective HIV risk reduction tools, such as regular testing, consistent condom usage, and PrEP are available, they remain underutilized among women who are unaware of their own risk and susceptibility to HIV and, thus, uncommitted to decreasing HIV risk. Addressing these barriers is essential to improving the uptake and effectiveness of HIV prevention strategies among women.

This study highlights the multi-faceted challenges women in the United States face in reducing their risk of HIV and underscores the need for community-informed interventions. Insights from WLWH reveal critical gaps in awareness of HIV susceptibility and the influence of personal and partner characteristics on HIV acquisition. Addressing these gaps requires interventions that increase HIV risk awareness, empower women to negotiate safer sex practices, and foster commitment to risk reduction. By incorporating education, skill-building, and peer-led support, future strategies can better address the structural, social, and behavioral barriers women face, ultimately enhancing the effectiveness of HIV prevention efforts for women.
